# Effects of Dietary Coated Folic Acid and Folic Acid Addition on Lactation Performance, Rumen Fermentation, and Hepatic Lipid Content in Early Lactation Dairy Cows

**DOI:** 10.3390/ani15020169

**Published:** 2025-01-10

**Authors:** Nan Sun, Songming Zou, Jiaxin Feng, Gang Guo, Qiang Liu, Yawei Zhang, Lei Chen, Wenjie Huo, Cong Wang

**Affiliations:** College of Animal Sciences, Shanxi Agricultural University, Taigu, Jinzhong 030801, China; s1317688430@163.com (N.S.); songmingabc@163.com (S.Z.); fengjiaxin5575@163.com (J.F.); guosteel1984@163.com (G.G.); ywzhang@sxau.edu.cn (Y.Z.); cl1016zj@126.com (L.C.); huohuo-1982@163.com (W.H.); wangdx0321@163.com (C.W.)

**Keywords:** folic acid, milk yield, rumen volatile fatty acids, hepatic lipid content, dairy cows

## Abstract

This study evaluated the influences of coated folic acid (CFA) and folic acid (FA) on lactation performance, apparent digestibility, rumen volatile fatty acid (VFA) production, blood metabolism, and hepatic lipid content in cows. A total of 140 Holstein cows were allocated to seven groups in a randomized block design. Cows in the control received no addition, those in the in low CFA (LCFA), medium CFA (MCFA), and high CFA (HCFA) groups received CFA at 135, 270, and 405 mg FA/d, and those in the low FA (LFA), medium FA (MFA), and high FA (HFA) groups received FA at 135, 270, and 405 mg/d. The experiment began 5 weeks before calving to 6 weeks after calving. When increasing the level of CFA, the fat-corrected milk (FCM), fat and protein yields, de novo fatty acid content, and feed efficiency increased linearly. A linear increase was observed for nutrient digestibility and ruminal total VFAs. The blood total protein, albumin, superoxide dismutase, glutathione peroxidase, and folate increased linearly, but blood non-esterified fatty acids and β-hydroxybutyric acid and hepatic lipids decreased linearly. When increasing the level of FA, the FCM and milk fat yields increased linearly, but the rumen total VFA increased quadratically. Compared with MFA, cows receiving MCFA had a greater milk yield and lower hepatic lipids. Overall, the addition of CFA increased the milk yield and decreased the hepatic lipid content in cows.

## Simple Summary

The mobilization of body tissue due to a lower feed intake causes more fatty acids enter into the liver, resulting in a decrease of the energy utilization efficiency and an increase of hepatic lipid content in cows during the early lactation period. An improvement in nutrient utilization efficiency and antioxidant status can cause more nutrient and energy to be partitioned to milk production. Folate is an essential nutrient for rumen cellulolytic bacteria growth and milk synthesis and has antioxidant properties. The present study showed that supplementing coated folic acid in cow diets increased the milk yield and decreased the hepatic lipid content in cows in early lactation. When supplementing the same level of folic acid, cows receiving coated folic acid had a similar rumen total of volatile fatty acid concentration, greater milk yield, and lower hepatic lipid content compared with those consuming folic acid. Therefore, coated folic acid supplementation is recommended in early lactation dairy cows.

## 1. Introduction

During the early lactation period, a negative nutrient balance causes a mobilization of body tissue in cows [1]. Thus, the lactation performance and immunity status decreases but blood β-hydroxybutyric acid (BHB) and non-esterified fatty acid (NEFA) and hepatic lipid contents increase in cows [1]. Folic acid (FA) transmits one carbon unit in the process of thymidylate, purine, and methionine synthesis and is essential in new cell production, antioxidant status maintenance, and lipid, protein, and energy metabolism in cows [2]. Studies have found that plasma folate concentration was negatively related to BHB and NEFA contents and was the lowest at 0 to 55 days in milk (DIM) [3,4]. In dairy cows, FA deficiency occurred when milk production was above 32 kg/d and the first consequence of deficiency was a loss of metabolic efficiency [2,5]. Due to a higher rumen degradability of FA (97%), only 2.5 to 4.5% of the ingested FA reached the portal blood in dairy cows [6,7]. Thus, coated FA (CFA) supplementation was recommended in early lactation cows [5,8].

Previous studies have found that dietary FA or CFA addition increased milk yield and decreased blood NEFA and BHB contents, but did not affect dry matter intake (DMI) and body weight (BW) during the early lactation of cows [8,9,10]. The increase in milk performance might be due to an improvement in antioxidant status and nutrient utilization efficiency, which could cause more nutrient and energy to be partitioned to milk production, resulting in a decrease in hepatic lipid content [11]. Folate has antioxidant properties [12,13]. El-Tarabany et al. [14] found that FA capsule addition increased blood total protein, albumin, glutathione peroxidase (GSH-Px), and superoxide dismutase (SOD) and decreased blood malondialdehyde (MDA) in early pregnancy ewes. Others have found that dietary CFA or FA addition improved immune status in early lactation cows [10,15,16]. However, limited research has evaluated the effect of CFA addition on antioxidant status in cows. Nonetheless, in vitro and in vivo studies have demonstrated that FA is an essential nutrient for rumen cellulolytic bacteria growth and the addition of FA or CFA increases the apparent digestibility and ruminal total volatile fatty acid (VFA) content [9,17,18]. A meta-analysis showed that the stimulatory effect of supplementary FA on rumen fermentation was a main reason for the increase of milk yield in cows [19]. Compared with the same level of FA addition, the ruminally available FA is less for CFA and might not meet the requirements of microbes. Thus, comparing the effects of CFA and FA on the ruminal VFA production and microbial populations in cows was necessary.

Based on the studies above, it was hypothesized that supplementing CFA in cow diets could increase lactation performance, improve antioxidant status, and decrease hepatic lipid content and that the coating of the FA would not impair the positive effects of the FA on rumen VFA production and microbial growth. Therefore, this study evaluated the dietary addition of three levels of CFA and three levels of FA from 5 weeks before calving on milk performance, apparent digestibility, rumen VFA production, antioxidant status, and hepatic lipid content in early lactation Holstein cows.

## 2. Materials and Methods

### 2.1. Cows and Treatments

The cows were cared for based on the guidelines of the Institutional Animal Care and Use Committee of Shanxi Agricultural University (SXAU-EAW-2023CO.MC.011029225) and were supplemented from 5 weeks before calving until 6 weeks post-calving. A total of 140 Holstein cows were blocked based on parity (2.03 ± 0.18), previous 305-day milk yield (10155 ± 256.2 kg), and BW (676 ± 33.6 kg) and then randomly allocated to 1 of 7 groups. Cows in the control received no FA or CFA addition, those in the low CFA (LCFA), medium CFA (MCFA), and high CFA (HCFA) groups received CFA at 135, 270, and 405 mg FA/d, and those in the low FA (LFA), medium FA (MFA), and high FA (HFA) groups received FA at 135, 270 and 405 mg/d. The supplementation dose of CFA or FA was determined according to the results of Zhang et al. [9], which showed that the dietary addition of CFA at 135 mg FA/d increased milk yield in early lactation cows. The CFA used in this study had 2% FA concentration and was produced by Shanxi Guomu Technology Co., Ltd. (Taiyuan, China). The release ratio of FA in the CFA was 68.4% in the intestine and 25.3% in the rumen, respectively. Thus, the ruminal FA supply in the LCFA, MCFA, and HCFA groups was 33.75, 67.5, and 101.25 mg/d, respectively. The total mixed ratio (TMR; Table 1) of prepartum and postpartum contained FA 0.31 and 0.34 mg/kg dry matter (DM), respectively, and was formulated based on the recommendations of NASEM [20].

Cows were housed in a 2-row head-to-head tie-stall barn fitted with individual feeding, fed twice (07:00 and 15:00) daily, allowing for 10% of refusals, and were milked at 06:00, 14:00, and 22:00 daily after calving. The water was free access for cows during the trial. Before the morning feeding, all cows consumed completely a mixture of 500 g TMR and FA or CFA by using the individual head-lock gates.

### 2.2. Data Collection, Sampling, and Determination

Data and samples were collected from each cow individually during 6 weeks postpartum. The BW of cows was recorded at 2 h after calving and at days 42 postpartum after the 06:00 milking and before the morning feeding. The daily TMR offered and refused were weighted, sampled weekly, and dried at 65 °C for DMI calculation. The daily milk yield was recorded from days 4 postpartum. Milk samples at the 06:00, 14:00, and 22:00 milkings were collected once weekly, polled by cow and day, and measured for protein, lactose, fat, and urea nitrogen concentrations by using a 133B infrared spectrophotometry (Foss Electric, Hillerød, Denmark). The 4% fat-corrected milk (FCM) yield was calculated by the following formula: 4% FCM = 0.4 × milk yield + 1.5 × milk fat yield [20]. Milk samples for the determination of fatty acids (FAs) composition were collected on days 40, 41, and 42 postpartum and stored at −20 °C. The FAs composition was analysed by using a gas chromatography (6890N, Agilent Technologies, Santa Clara, CA, USA) based on reports of Shingfield et al. [21].

The total-tract digestibility was estimated by collecting rectum faecal samples (150 g) on days 40, 41, and 42 postpartum with an 8 h interval. The samples of faeces were pooled by cow and dried at 65 °C. The air-dried samples of TMR and faeces were ground to pass through a 1 mm sieve and determined for DM (934.01), organic matter (OM, 942.05), crude protein (CP, 988.05), and acid detergent fibre (ADF, 73.18) based on AOAC [22]. The measure of neutral detergent fibre (NDF) was done according to the reports of Van Soest et al. [23]. The concentrations of acid-insoluble ash in TMR or faeces were used as an internal marker to calculate digestibility and were measured according to the reports of Furuichi and Takahashi [24].

Ruminal fluid samples were taken by using a stomach tube at around 9:00 of days 14, 21, and 42 postpartum. The first 200 mL of fluid collected was abandoned and the subsequent 200 mL was strained using 4 layers of cheesecloth and determined for pH by a pH meter (TP110, TIMEPOWER Corp., Beijing, China). Fluid samples were stored at −20 °C for the determination of VFA and at −80 °C for the measurement of microbial copies. The VFA was measured using the internal standard method (2-ethylbutyric acid) and a GC9720 (Zhejiang Fuli Analytical Instruments Co., Ltd., Wenling, China). The chromatographic conditions were as follows: initial column temperature 70 °C, program heating; injector temperature 220 °C, injection volume 2 µL, splitting ratio 6; detector temperature 220 °C, tail blow 40 mL/min, hydrogen flow rate 35 mL/min, and air flow rate 350 mL/min. The microbial total DNA in the ruminal fluid was extracted by using the TIANamp stool DNA isolation kit (Tiangen Biotech Co., Ltd., Beijing, China) and then determined for quantity and quality by using a spectrophotometer (NanoDrop 2000, Thermo Scientific, Waltham, MA, USA) and agarose gel electrophoresis, respectively. The primer sets (Table 2) used for amplifying the 16S/18S rRNA genes of microbes were commercially synthesized (BGI Life Tech Co., Ltd., Beijing, China) and specificities were checked by a primer-BLAST search of GenBank. The sample-derived DNA standards for qPCR assay were produced by regular PCR, purified using the PureLinkTM Quick Gel Extraction and PCR Purification Combo Kit (Beijing Bioteke Biotechnology Co. Ltd., Beijing, China), and quantified by a spectrophotometer [25]. The products of PCR were diluted in a ten-fold gradient using nuclease-free water to establish the standard curves of the target microbes. The qPCR assay was performed in a StepOne Plus™ real-time PCR system (Applied Biosystems, Foster City, CA, USA) and using a TB GreenTM Premix Ex TaqTM ⅡKIT (Takara, Beijing, China). The reaction system and qPCR assay conditions were the same as those in the reports of Zhang et al. [9] and the copies of microbes were calculated based on the reports of Metzler-Zebeli et al. [26].

Blood samples were collected via the coccygeal vein into a tube containing sodium heparin and a tube containing sodium fluoride at approximately 9:00 of days 14, 21, and 42 postpartum, centrifuged at 4 °C and 2500× *g* for 15 min to obtain plasma and serum, and then stored at −20 °C. Based on the instructions of commercially available kits (Shanghai Fusheng Industrial Co., Ltd., Shanghai, China), the glucose, total protein, albumin, SOD, GSH-Px, MDA, folate, 5-methyltetrahydrofolate (5-MTHF), homocysteine (Hcy), triglyceride (TG), BHB, very low density lipoprotein (VLDL), and apoliprotein B100 (ApoB100) were measured using a Hitachi 7600 automated biochemistry analyser (Hitachi Co., Ltd., Tokyo, Japan) and NEFA was analysed using a spectrophotometer (Yipu Co., Ltd., Shanghai, China).

Liver samples of approximately 1.5 g of wet weight were obtained from each cow after blood sampling on days 56 postpartum via percutaneous biopsy as reported by Gross et al. [27], snap-frozen in liquid nitrogen, and subsequently kept at −80 °C. The hepatic total lipid was analysed gravimetrically [28] and TG and folate were measured by using commercially available kits (Shanghai Fusheng Industrial Co., Ltd., China).

### 2.3. Statistical Analyses

The feed efficiency of each cow was calculated as the milk yield (actual yield and FCM) divided by DMI. All data were analysed statistically by using the MIXED procedure of SAS (version 9.3, SAS Institute Inc., Cary, NC, USA). The repeated measures model contained fixed effects of week, treatment, and the interaction of treatment and week and the random effect of cow. The normality and homoscedasticity of the distributions were checked by Shapiro–Wilk and Bartlett tests. When needed, variables were normalized using the Box–Cox transformation. Contrasts were constructed to examine treatment effects, the CFA and FA supplemented level, with orthogonal polynomials accounting for the unequal spacing of CFA and FA dosage levels, and FA source (MCFA vs. MFA). Tukey’s multiple range test was used for the evaluation of differences between the groups. Degrees of freedom were determined according to the Kenward–Roger method. Data are presented as the least squares mean and standard error of the mean. The level of statistical significance is expressed at *p* < 0.05.

## 3. Results

### 3.1. DMI, BW, and Lactation Performance

As shown in Table 3, the DMI, BW at days 0 and 42 postpartum, and body weight change (BWC) were not affected by CFA or FA addition. The actual milk, FCM, and milk protein and fat yields increased linearly (*p* < 0.05) and the lactose yield and milk fat, protein, lactose, and urea nitrogen contents were unaltered with increasing CFA addition. The milk de novo FAs content linearly increased (*p* < 0.05), mixed FAs was unaltered, and preformed FAs linearly decreased (*p* = 0.043) with increasing CFA supply. A linear increase (*p* = 0.035) in feed efficiency was observed with increasing CFA addition. However, the milk, lactose, and protein yields, milk component content, FAs composition, and feed efficiency were unaltered and only the FCM and milk fat yields linearly increased (*p* < 0.05) when increasing the FA supply. Compared with MFA addition, the milk and milk protein yields, feed efficiency, and milk preformed FAs content were greater (*p* < 0.05) and milk de novo FAs content was lower (*p* < 0.012) in cows consuming MCFA.

### 3.2. Nutrient Apparent Digestibility

The apparent digestibility of DM, OM, CP, NDF, and ADF linearly increased (*p* < 0.05) with increasing CFA addition (Table 4). However, DM digestibility quadratically increased (*p* = 0.037), OM and NDF digestibility linearly increased (*p* < 0.05), and CP and ADF digestibility were unaltered with increasing FA addition. No significant difference was found in apparent digestibility between MCFA and MFA addition.

### 3.3. Ruminal Fermentation and Microbial Population

The rumen pH and acetate, butyrate, valerate, and isobutyrate proportions were not affected by CFA or FA supply (Table 5). The total VFA content, isovalerate proportion, and acetate to propionate ratio linearly increased (*p* < 0.05) but the propionate proportion was unchanged with increasing CFA addition. However, the total VFA content quadratically increased (*p* = 0.041), propionate and isovalerate proportions linearly increased (*p* < 0.05), and acetate to propionate ratio was unaltered with increasing FA addition. The total VFA concentration was similar but acetate to propionate ratio and propionate and isovalerate proportions were greater (*p* < 0.05) for cows consuming MCFA compared with those consuming MFA.

The population of *Butyrivibrio fibrisolvens* was not affected by CFA or FA addition. The populations of rumen microbes except for *B. fibrisolvens* linearly increased (*p* < 0.05) when increasing the CFA supply. Nevertheless, the populations of total bacteria, protozoa, *Fibrobacter fuccinogenes*, *Ruminobacter amylophilus*, and *Prevotella ruminicola* linearly increased (*p* < 0.05) and fungi, *Ruminococcus albus*, and *R. flavefaciens* quadratically increased (*p* < 0.05) with increasing FA supply. The populations of protozoa, *F. fuccinogenes*, and *P. ruminicola* were lower (*p* < 0.05) in cows consuming MCFA than in those consuming MFA.

### 3.4. Blood Metabolites and Hepatic Lipid Content

As shown in Table 6, the blood glucose, TG, VLDL, and ApoB100 contents were unaltered with increasing CFA addition. Blood total protein, albumin, SOD, GSH-Px, folate, and 5-MTHF contents and hepatic folate content linearly increased (*p* < 0.05) and blood MDA, Hcy, NEFA, and BHB contents and hepatic total lipid and TG contents linearly decreased (*p* < 0.05) when increasing the CFA supply. Nevertheless, dietary FA addition did not affect blood metabolites and hepatic lipid, TG, and folate contents.

## 4. Discussion

The aim of this study was to evaluate the effects of dietary CFA or FA addition on the lactation performance, antioxidant status, and hepatic lipid content in cows of early lactation. The regulation of FA in rumen fermentation plays a major role in improving lactation performance in cows [19]. However, only 25.3% of the FA in the CFA is released in the rumen and the coating might impair the positive influences of FA on rumen fermentation. Thus, the effects of CFA or FA addition were evaluated and the comparison of the effects of CFA and FA addition was done in the current study.

### 4.1. Effect of CFA Addition

In line with the results of previous studies [8,9], the DMI of cows was unchanged with CFA addition. The limited response of BW and BWC were consistent with the results of Zhang et al. [9], indicating that dietary CFA supply did not decrease body reserve mobilization in early lactation cows. The increase in milk and milk component yields as well as feed efficiency might result from the improvement in milk synthesis, antioxidant status, and rumen VFA production. Folate and folate receptor α are essential factors for milk protein synthesis in cows [29]. The increase of blood albumin level and SOD and GSH-Px activities indicated that immunity and antioxidant status were improved in cows receiving CFA addition, and this would cause more energy partitioned to milk production [11]. In addition, the increase of rumen total VFA production also could cause more substrates and energy to be partitioned to milk synthesis [30]. Furthermore, the increase in milk de novo FAs as well as the decrease in preformed FAs indicated that addition of CFA promoted milk fat synthesis and were in line with the increase of rumen acetate concentration as well as the decrease of blood NEFA concentration. Milk de novo FAs originate mainly from acetate and about 40% of blood NEFAs is used as a source of milk preformed FAs during the early lactation period of cows [31,32]. Urrutia and Harvatine observed that milk, milk fat, and de novo FAs yields increased with an increasing concentration of rumen acetate in early lactation cows [33]. Similarly, previous studies found that dietary supplementation with FA or CFA 3 weeks before calving increased milk yield during the early lactation period in cows [8,9,10].

The increase in apparent digestibility was in line with the results of a previous study [9] and was associated with an enhancement of nutrient degradation in the rumen, reflected as the increase of rumen total VFA content. Folate is an essential nutrient for rumen microbial growth and plays a major role in maintaining the structure and function of the digestive tract [17,34]. The CFA used in the present study released 25.3% of its FA in the rumen and 68.4% in the intestine and thus had the potential to stimulate nutrient digestion both in the rumen and in the intestine. Wang et al. [35] reported that ruminal DM, CP, and NDF degradability increased with dietary CFA addition in steers. Parnian-Khajehdizaj et al. [18] found that total-tract and ruminal DM digestibility increased with the addition of FA in vitro.

The mean pH of ruminal fluid was 6.45 for cows without or with CFA addition and was suitable for bacterial growth and nutrient degradation [36]. A significant change was not observed in rumen pH, but total VFA content increased for the CFA supply groups. The increase in rumen total VFA content and the acetate to propionate ratio were associated with the increase in populations of bacteria, fungi, and protozoa, indicating that FA addition was necessary for microbial growth, especially for those responsible for fibre digestion. More specifically, the increase of acetate production was associated with positive changes in the populations of *R. albus*, *R. flavefaciens*, and *F. succinogenes* and the increase of propionate production was consistent with an increase in the populations of *P. ruminicola* and *Rb. amylophilus*, which are responsible for starch degradation [37]. Folate is required in microbial DNA and protein synthesis [2,17]. The stimulatory effects of CFA on cellulolytic bacteria populations and fibre degradation would result in an increase in substrate for other microbial growth [38]. Likewise, an in vitro study found that rumen total VFA concentration at 12 or 24 h of incubation increased with FA addition [18]. Zhang et al. [9] observed that dietary CFA addition increased rumen VFA concentrations and microbial populations in cows. Moreover, stimulatory effects of FA on gut microbiota and VFA production were observed in monogastric animals [39].

The limited response of blood glucose concentration was in line with the report of Zhang et al. [9]. The positive changes in blood total protein and albumin concentrations were associated with the increase of CP digestibility, indicating that dietary N-utilization efficiency was improved in cows receiving a CFA addition. Blood albumin is synthesized in the liver and decreases during inflammation, SOD and GSH-Px are key enzymes of the antioxidant system, and MDA is a product of lipid peroxidation [1,40]. Therefore, the increase in blood albumin level and GSH-Px and SOD activities as well as the reduction in MDA level showed that immunity and antioxidant status were improved and were associated with the decrease in blood Hcy and the increase in blood 5-MTHF for cows receiving a CFA addition. Hcy is derived from the process of methionine demethylation and can stimulate lipid peroxidation by generating partially reduced reactive oxygen species [12,13]. However, 5-MTHF is able to decrease the level of intracellular reactive oxygen species [12,13]. Likewise, Khan et al. [10] and Lopreiato et al. [16] reported that the immunity status of peripartal cows was improved by CFA or FA addition. El-Tarabany et al. [14] found that dietary FA supplementation increased blood total protein, albumin, SOD, and GSH-Px and decreased MDA in early pregnancy ewes. Blood NEFA from adipose tissue mobilization can be utilized to synthesize milk fat or enter into the liver [41,42]. In the liver, NEFA is completely oxidized to supply energy, partially oxidized to BHB, or stored as TG which also can be exported in the form of VLDL [41,42]. The limited responses of blood VLDL and ApoB100 concentrations suggested that CFA addition did not affect the export of hepatic lipid. Since BW and BWC were unaltered and milk fat yield increased for cows receiving CFA addition, the reduction in blood NEFA and BHB concentrations were probably due to the fact that more blood NEFA was used as a source of milk fat synthesis, and this might be a reason of the reduction in hepatic total lipid and TG contents in cows with CFA addition. The regulation of folate or 5-MTHF on hepatic immunity and energy metabolism was probably another reason for the decrease in hepatic total lipid and TG contents. Studies have verified that the addition of FA attenuated oxidative stress and lipid deposition in the livers of mice [43,44]. Likewise, Duplessis et al. [4] found that blood BHB and NEFA levels were negatively related to the level of folate in cows during the transition period. However, other studies found that hepatic total lipid or TG contents were unaltered with a weekly injection of FA 160 mg or increased with dietary supplementation with FA 2.6 g/d in early lactation cows [8,45]. The divergent results might be associated with the different modes of FA addition in these studies. The addition of FA by injection had a limited effect on nutrient digestibility and only 2.5 to 4.5% of the supplementary FA could reach the portal blood in the cows [6,7,19].

### 4.2. Effect of FA Addition

The dietary addition of FA had no influence on DMI, BWC, and blood and hepatic folate contents. Thus, the positive change of FCM and milk fat yields was probably due to the increase of rumen total VFA content as well as total-tract DM, OM, and NDF digestibility. Likewise, Wang et al. [19] reported that the regulation of supplementary FA on rumen fermentation was an important reason for the increase in milk performance in cows. When increasing the level of FA addition, a quadratic response of rumen total VFA content was observed and was in line with the change of fungi, *R. flavefaciens*, and *R. albus* populations, showing that higher levels of ruminal FA supply were not required for cellulolytic microbial growth.

### 4.3. Comparison of CFA and FA Addition

When compared with cows receiving MFA addition, a significant difference was not found on rumen total VFA content and total-tract nutrient digestibility for cows consuming MCFA. However, greater milk and milk protein yields and lower hepatic lipid and TG contents were observed in cows receiving MCFA addition. The results suggested that dietary supplementation of CFA should be recommended in cows. In addition, the rumen propionate molar proportion and protozoa, *F. fuccinogenes*, and *P. ruminicola* populations were greater and the acetate to propionate ratio was lower for cows consuming MFA than those receiving MCFA, indicating that a higher level of ruminal FA supply was required for propionate production.

## 5. Conclusions

Dietary CFA addition begun 5 weeks before calving increased the milk yield and decreased the blood NEFA and BHB and hepatic lipid contents in cows of early lactation, and this might be associated with the properties of folate in improving nutrient utilization and antioxidant status. The addition of CFA might cause more nutrient and energy to be partitioned to milk synthesis. The dietary addition of FA increased FCM and milk fat yields, showing that the regulation of FA on rumen fermentation was one of the main reasons for the improvement in milk performance in the cows. The dietary addition of FA did not improve antioxidant status or decrease hepatic lipid content in cows and the ruminal total VFA production was not affected by the coating of FA. Thus, the CFA supplement is recommended in cows.

## Figures and Tables

**Table 1:** Ingredients and chemical composition of the basal diets (DM%).

Ingredients	Prepartum Diet	Postpartum Diet
Corn silage	36.0	26.0
Alfalfa hay		13.0
Oat hay	24.0	11.0
Corn grain, ground	15.1	24.0
Wheat bran	6.0	6.0
Soybean meal	10.0	10.6
Rapeseed meal	2.4	2.5
Cottonseed cake	5.2	5.0
Calcium carbonate	0.7	0.6
Salt	0.6	0.5
Dicalcium phosphate	0.4	0.3
Mineral and vitamin premix ^1^	0.6	0.5
Chemical composition		
Organic matter	94.5	94.5
Crude protein	16.0	17.2
Ether extract	3.2	3.2
Neutral detergent fibre	35.3	31.1
Acid detergent fibre	21.5	19.3
Calcium	0.72	0.73
Phosphorus	0.47	0.48
Folate, mg/kg	0.31	0.33
Net energy for lactation ^2^, MJ/kg	6.41	6.63

^1^ Contained per kg premix: 20,000 mg Fe, 1600 mg Cu, 8000 mg Mn, 7500 mg Zn, 1.20 mg I, 60 mg Se, 20 mg Co, 820,000 IU vitamin A, 300,000 IU vitamin D, and 10,000 IU vitamin E. ^2^ Estimated based on NASEM [20].

**Table 2:** PCR primers of microbes for real-time PCR assay.

Target Species	Primer Sequence (5′-3′)	GenBank Accession No.	Annealing Temperature (°C)	Size (bp)
Total bacteria	F: CGGCAACGAGCGCAACCC R: CCATTGTAGCACGTGTGTAGCC	CP058023.1	60	147
Total fungi	F: GAGGAAGTAAAAGTCGTAACAAGGTTTC R: CAAATTCACAAAGGGTAGGATGATT	GQ355327.1	57.5	120
Total protozoa	F: GCTTTCGWTGGTAGTGTATT R: CTTGCCCTCYAATCGTWCT	HM212038.1	59	234
*R*. *albus*	F: CCCTAAAAGCAGTCTTAGTTCG R: CCTCCTTGCGGTTAGAACA	CP002403.1	60	176
*R. flavefaciens*	F: ATTGTCCCAGTTCAGATTGC R: GGCGTCCTCATTGCTGTTAG	AB849343.1	60	173
*B.* *fibrisolvens*	F: ACCGCATAAGCGCACGGA R: CGGGTCCATCTTGTACCGATAAAT	HQ404372.1	61	65
*F. succinogenes*	F: GTTCGGAATTACTGGGCGTAAA R: CGCCTGCCCCTGAACTATC	AB275512.1	61	121
*Rb. amylophilus*	F: CTGGGGAGCTGCCTGAATG R: GCATCTGAATGCGACTGGTTG	MH708240.1	60	102
*P. ruminicola*	F: GAAAGTCGGATTAATGCTCTATGTTG R: CATCCTATAGCGGTAAACCTTTGG	LT975683.1	58.5	74

**Table 3:** Effects of coated folic acid and folic acid on dry matter intake, body weight, and lactation performance in early lactation cows.

Item ^1^	Treatments ^2^							SEM	Contrast, *p* ^3^					
	Control	CFA			FA				Treatment	Source	CFA Level		FA Level	
		LCFA	MCFA	HCFA	LFA	MFA	HFA				Linear	Quadratic	Linear	Quadratic
Dry matter intake, kg/d	26.1	25.6	25.3	25.5	25.5	26.1	25.8	0.551	0.301	0.432	0.265	0.332	0.158	0.316
Body weight days 0 postpartum, kg	665	672	681	667	677	679	664	21.08	0.453	0.668	0.556	0.437	0.331	0.365
Body weight days 42 postpartum, kg	636	648	660	647	651	651	633	19.45	0.378	0.346	0.477	0.356	0.273	0.254
Body weight change, kg/d	−0.69	−0.57	−0.50	−0.48	−0.61	−0.66	−0.74	0.164	0.096	0.371	0.083	0.172	0.113	0.258
Milk yield														
Actual milk, kg/d	41.0 ^b^	42.9 ^a^	42.8 ^a^	44.3 ^a^	40.6 ^b^	41.3 ^b^	39.8 ^b^	0.947	0.025	0.014	0.039	0.234	0.351	0.172
Fat-corrected milk, kg/d	41.7 ^c^	44.1 ^ab^	44.3 ^ab^	45.6 ^a^	43.3 ^b^	43.7 ^b^	41.7 ^c^	1.069	0.039	0.076	0.008	0.118	0.042	0.098
Fat, kg/d	1.69 ^b^	1.80 ^a^	1.81 ^a^	1.86 ^a^	1.80 ^a^	1.81 ^a^	1.72 ^b^	0.045	0.028	0.124	0.013	0.274	0.022	0.107
Protein, kg/d	1.35 ^b^	1.40 ^ab^	1.45 ^a^	1.51 ^a^	1.32 ^b^	1.37 ^b^	1.37 ^b^	0.034	0.033	0.018	0.011	0.135	0.157	0.263
Lactose, kg/d	2.16	2.27	2.28	2.35	2.12	2.16	2.09	0.093	0.108	0.236	0.096	0.318	0.263	0.332
Milk composition content														
Fat, %	4.12	4.18	4.24	4.20	4.44	4.38	4.32	0.113	0.116	0.254	0.108	0.262	0.253	0.174
Protein, %	3.28	3.26	3.38	3.41	3.24	3.32	3.45	0.093	0.084	0.118	0.253	0.304	0.275	0.311
Lactose, %	5.26	5.29	5.33	5.31	5.22	5.24	5.26	0.103	0.515	0.172	0.233	0.139	0.216	0.158
Urea nitrogen, mg/dL	12.2	12.0	11.9	11.7	11.5	12.3	12.5	0.191	0.346	0.534	0.382	0.178	0.333	0.264
De novo FAs, g/100 g FAs	25.3 ^b^	26.6 ^a^	26.9 ^a^	27.0 ^a^	24.8 ^b^	24.7 ^b^	25.1 ^b^	0.402	0.013	0.012	0.043	0.214	0.122	0.333
Mixed FAs, g/100 g FAs	31.2	30.8	30.7	30.9	31.3	31.9	31.3	0.731	0.476	0.273	0.118	0.154	0.237	0.134
Preformed FAs, g/100 g FAs	42.2 ^a^	41.5 ^ab^	41.0 ^b^	40.9 ^b^	42.5 ^a^	42.0 ^a^	41.9 ^a^	0.392	0.011	0.033	0.024	0.315	0.245	0218
Feed efficiency	1.57 ^b^	1.68 ^ab^	1.71 ^a^	1.73 ^a^	1.59 ^b^	1.58 ^b^	1.54 ^b^	0.062	0.008	0.026	0.035	0.277	0.412	0.254

^1^ Fat-corrected milk = 0.4 × milk yield + 15 × fat yield; FAs = fatty acids; De novo FAs are defined as FAs with chain length < 16 carbons, preformed FAs are defined as FAs with chain length > 16 carbons, and mixed FAs are defined as C16:0 plus C16:1 cis-9 and iso-C16:0). ^2^ Control: without CFA or FA addition; LCFA, MCFA, and HCFA with addition of CFA 135, 270, and 405 mg FA/d, respectively; LFA, MFA, and HFA with addition of FA 135, 270, and 405 mg/d, respectively. ^3, a, b, c^ Means with different superscripts in each row differ significantly (*p* < 0.05); Treatment: the difference between the overall treatments; Source: a contrast of MCFA and MFA; CFA or FA level: orthogonal polynomials for response due to different levels of CFA or FA addition.

**Table 4:** Effects of coated folic acid and folic acid supplementation on total tract nutrient digestibility in early lactation cows.

Item	Treatments ^1^							SEM	Contrast, *p* ^2^					
	Control	CFA			FA				Treatment	Source	CFA Level		FA Level	
		LCFA	MCFA	HCFA	LFA	MFA	HFA				Linear	Quadratic	Linear	Quadratic
Dry matter, %	71.3 ^c^	73.4 ^b^	75.5 ^a^	75.0 ^a^	73.7 ^b^	73.8 ^b^	70.7 ^c^	1.077	0.014	0.103	0.008	0.535	0.691	0.037
Organic matter, %	72.0 ^c^	74.8 ^b^	76.3 ^a^	76.7 ^a^	74.5 ^ab^	74.3 ^b^	73.6 ^b^	1.135	0.017	0.093	0.002	0.832	0.048	0.136
Crude protein, %	69.4 ^b^	70.8 ^b^	72.1 ^a^	72.6 ^a^	70.8 ^b^	71.3 ^b^	68.2 ^b^	1.136	0.014	0.116	0.017	0.555	0.334	0.722
Neutral detergent fibre, %	53.2 ^c^	56.2 ^b^	56.3 ^b^	59.0 ^a^	58.3 ^a^	57.2 ^a^	58.0 ^a^	1.408	0.007	0.374	0.011	0.924	0.014	0.198
Acid detergent fibre, %	49.9 ^b^	50.9 ^b^	53.6 ^a^	53.4 ^a^	51.5 ^b^	50.7 ^b^	51.4 ^b^	1.382	0.018	0.105	0.034	0.797	0.463	0.566

^1^ Control: without CFA or FA addition; LCFA, MCFA, and HCFA with addition of CFA 135, 270, and 405 mg FA/d, respectively; LFA, MFA, and HFA with addition of FA 135, 270, and 405 mg/d, respectively. ^2, a, b, c^ Means with different superscripts in each row differ significantly (*p* < 0.05); Treatment: the difference between the overall treatments; Source: a contrast of MCFA and MFA; CFA or FA level: orthogonal polynomials for response due to different levels of CFA or FA addition.

**Table 5:** Effects of coated folic acid and folic acid supplementation on rumen fermentation and microbial population in early lactation cows.

Item ^1^	Treatments ^2^							SEM	Contrast, *p* ^3^					
	Control	CFA			FA				Treatment	Source	CFA Level		FA Level	
		LCFA	MCFA	HCFA	LFA	MFA	HFA				Linear	Quadratic	Linear	Quadratic
Rumen pH	6.56	6.51	6.46	6.40	6.35	6.29	6.41	0.133	0.239	0.246	0.128	0.117	0.281	0.121
Total VFA, mmol/L	112 ^c^	121 ^b^	126 ^ab^	132 ^a^	129 ^a^	134 ^a^	112 ^c^	2.459	0.028	0.286	0.033	0.187	0.241	0.041
Acetate, %	61.8	62.9	62.4	62.4	62.8	60.9	61.4	1.245	0.133	0.177	0.168	0.259	0.187	0.233
Propionate, %	22.9 ^b^	21.9 ^b^	22.2 ^b^	22.2 ^b^	22.9 ^b^	25.1 ^a^	24.5 ^a^	1.122	0.047	0.031	0.447	0.696	0.027	0.251
Butyrate, %	10.3	9.7	9.7	9.5	9.00	8.81	9.00	0.863	0.663	0.275	0.326	0.257	0.125	0.383
Valerate, %	2.50	2.50	2.53	2.75	2.35	2.55	2.32	0.042	0.236	0.138	0.241	0.287	0.129	0.286
Isobutyrate, %	1.33	1.18	1.35	1.21	1.25	1.12	1.09	0.013	0.106	0.096	0.253	0.426	0.188	0.167
Isovalerate, %	1.11 ^c^	1.77 ^b^	1.73 ^b^	1.96 ^a^	1.60 ^b^	1.52 ^b^	1.64 ^b^	0.038	0.040	0.019	0.027	0.159	0.042	0.246
Acetate/Propionate	2.70 ^b^	2.87 ^a^	2.81 ^a^	2.81 ^a^	2.74 ^ab^	2.43 ^b^	2.51 ^b^	0.006	0.032	0.028	0.017	0.263	0.058	0.389
Total bacteria, ×10^11^/mL	5.55 ^c^	8.16 ^b^	8.47 ^b^	9.30 ^a^	9.14 ^a^	9.13 ^a^	7.89 ^b^	0.200	0.003	0.343	0.018	0.604	0.034	0.529
Fungi, ×10^7^/mL	2.51 ^c^	3.70 ^b^	4.90 ^a^	5.12 ^a^	5.24 ^a^	5.35 ^a^	2.76 ^c^	0.232	0.007	0.312	0.020	0.349	0.306	0.017
Protozoa, ×10^5^/mL	1.31 ^c^	1.53 ^c^	2.81 ^b^	3.61 ^a^	3.50 ^a^	3.54 ^a^	3.69 ^a^	0.146	0.001	0.014	0.008	0.200	0.007	0.176
*R. albus*, ×10^8^/mL	1.06 ^c^	1.12c	1.66 ^b^	2.60 ^a^	2.40 ^a^	1.72 ^b^	0.97 ^c^	0.155	0.032	0.651	0.006	0.118	0.104	0.003
*R. flavefaciens*, ×10^9^/mL	1.46 ^c^	2.36 ^b^	2.57 ^b^	3.03 ^b^	3.97 ^a^	3.13 ^ab^	1.65 ^c^	0.114	0.014	0.083	0.013	0.183	0.171	0.013
*F. fuccinogenes*, ×10^10^/mL	4.96 ^c^	5.17 ^b c^	5.97 ^b^	6.91 ^a^	7.34 ^a^	7.14 ^a^	7.25 ^a^	0.157	0.020	0.007	0.028	0.426	0.022	0.142
*B. fibrisolvens*, ×10^9^/mL	2.15	2.44	2.36	2.46	3.04	2.53	2.75	0.216	0.141	0.166	0.133	0.168	0.411	0.148
*P. ruminicola*, ×10^9^/mL	5.09 ^b^	5.48 ^b^	6.57 ^a^	6.77 ^a^	7.09 ^a^	6.76 ^a^	7.07 ^a^	0.211	0.007	0.023	0.029	0.253	0.016	0.432
*Rb. amylophilus*, ×10^8^/mL	2.94 ^b^	3.15 ^b^	3.55 ^a^	3.74 ^a^	3.68 ^a^	3.64 ^a^	3.61 ^a^	0.158	0.009	0.421	0.047	0.469	0.005	0.333

^1^ VFA = volatile fatty acid. ^2^ Control: without CFA or FA addition; LCFA, MCFA, and HCFA with addition of CFA 135, 270, and 405 mg FA/d, respectively; LFA, MFA, and HFA with addition of FA 135, 270, and 405 mg/d, respectively. ^3, a, b, c^ Means with different superscripts in each row differ significantly (*p* < 0.05); Treatment: the difference between the overall treatments; Source: a contrast of MCFA and MFA; CFA or FA level: orthogonal polynomials for response due to different levels of CFA or FA addition.

**Table 6:** Effects of coated folic acid and folic acid supplementation on blood metabolites and hepatic lipid and folate contents in early lactation cows.

Item ^1^	Treatments ^2^							SEM	Contrast, *p* ^3^					
	Control	CFA			FA				Treatment	Source	CFA Level		FA Level	
		LCFA	MCFA	HCFA	LFA	MFA	HFA				Linear	Quadratic	Linear	Quadratic
Blood														
Glucose, mmol/L	3.59	3.67	3.99	3.89	3.55	3.62	3.84	0.246	0.057	0.343	0.406	0.337	0.286	0.382
Total protein, g/L	73.9 ^b^	80.4 ^a^	77.5 ^a^	77.1 ^a^	73.6 ^b^	72.1 ^b^	70.8 ^b^	2.270	0.045	0.032	0.026	0.486	0.340	0.214
Albumin, g/L	40.5 ^b^	44.4 ^a^	43.4 ^a^	44.2 ^a^	40.7 ^b^	39.8 ^b^	40.6 ^b^	2.414	0.035	0.018	0.009	0.545	0.395	0.248
SOD, U/mL	75.5 ^b^	96.9 ^a^	101.6 ^a^	107.2 ^a^	81.3 ^b^	77.6 ^b^	72.1 ^b^	5.307	0.038	0.027	0.014	0.751	0.235	0.676
GSH-Px, U/mL	526 ^b^	585 ^a^	552 ^a^	570 ^a^	537 ^b^	529 ^b^	516 ^b^	18.41	0.003	0.011	0.006	0.521	0.156	0.273
MDA, nmol/L	6.41 ^a^	5.13 ^b^	5.50 ^b^	5.20 ^b^	5.96 ^ab^	6.31 ^a^	6.29 ^a^	0.431	0.048	0.037	0.027	0.382	0.086	0.337
Folate, µmol/L	13.7 ^b^	15.1 ^a^	15.9 ^a^	16.8 ^a^	13.9 ^b^	14.3 ^b^	14.5 ^b^	0.621	0.003	0.007	0.003	0.138	0.252	0.371
5-MTHF, ng/mL	27.6 ^b^	31.8 ^a^	31.9 ^a^	33.6 ^a^	27.9 ^b^	28.3 ^b^	28.3 ^b^	1.154	0.002	0.038	0.021	0.137	0.256	0.146
Hcy, µmol/L	11.7 ^a^	9.04 ^b^	9.78 ^b^	9.66 ^b^	11.2 ^a^	11.0 ^a^	11.9 ^a^	0.424	0.003	0.042	0.019	0.357	0.168	0.346
TG, mmol/L	7.39	7.18	7.23	7.09	7.16	7.33	7.10	0.108	0.381	0.287	0.395	0.468	0.476	0.648
NEFA, mmol/L	0.42 ^a^	0.38 ^b^	0.36 ^b^	0.36 ^b^	0.43 ^a^	0.41 ^a^	0.41 ^a^	0.019	0.027	0.005	0.016	0.677	0.482	0.575
BHB, mmol/L	1.06 ^a^	0.83 ^b^	0.88 ^b^	0.89 ^b^	0.96 ^a^	0.99 ^a^	1.01 ^a^	0.012	0.027	0.013	0.011	0.248	0.328	0.261
VLDL, mmol/L	1.11	1.14	1.17	1.17	1.19	1.16	1.14	0.452	0.100	0.873	0.459	0.593	0.431	0.647
ApoB100, pg/mL	1019	1035	1061	1041	1049	1027	1022	22.08	0.384	0.267	0.185	0.153	0.124	0.284
Liver														
Total lipid, mg/g fresh tissue	51.4 ^a^	46.5 ^b^	46.1 ^b^	45.6 ^b^	49.9 ^a^	51.8 ^a^	51.4 ^a^	0.825	0.022	0.006	0.013	0.421	0.332	0.286
TG, mg/g fresh tissue	23.4 ^a^	21.9 ^b^	21.3 ^b^	20.8 ^b^	22.3 ^a^	22.8 ^a^	23.5 ^a^	0.905	0.044	0.014	0.026	0.376	0.263	0.417
Folate, µg/g fresh tissue	8.12 ^b^	11.1 ^a^	11.1 ^a^	12.3 ^a^	8.27 ^b^	8.63 ^b^	8.07 ^b^	0.798	0.001	0.023	0.008	0.299	0.431	0.345

^1^ SOD = superoxide dismutase; GSH-Px = glutathione peroxidase; MDA = malondialdehyde; 5-MTHF= 5-methyltetrahydrofolate; Hcy = homocysteine; TG = triglyceride; NEFAs = non-esterified fatty acids; BHB = β hydroxybutyric acid; VLDL = very low-density lipoprotein; ApoB100 = apoliprotein B100. ^2^ Control: without CFA or FA addition; LCFA, MCFA, and HCFA with addition of CFA 135, 270, and 405 mg FA/d, respectively; LFA, MFA, and HFA with addition of FA 135, 270, and 405 mg/d, respectively. ^3, a, b^ Means with different superscripts in each row differ significantly (*p* < 0.05); Treatment: the difference between the overall treatments; Source: a contrast of MCFA and MFA; CFA or FA level: orthogonal polynomials for response due to different levels of CFA or FA addition.

## Data Availability

The data presented in this study are available on request from the corresponding author.
